# How to Design Greenway on Urban Land Utilization: Linking Place Preference, Perceived Health Benefit, and Environmental Perception

**DOI:** 10.3390/ijerph192013640

**Published:** 2022-10-20

**Authors:** Weiting Shan, Chunliang Xiu, Yining Meng

**Affiliations:** Northeastern University, Shenyang 110167, China

**Keywords:** land utilization, green space planning, place preference, multilevel mapping model, healthy city, greenway

## Abstract

The rapid urbanization and over-crowded urban environment have caused a serious public health crisis. Numerous studies have found that public green spaces can benefit human health and well-being. Therefore, a short supply or an inappropriate planning of public green spaces would exaggerate the health crisis. For all these reasons, how to create health-promoting greenways in urban areas becomes a critical and pressing challenge for urban sustainability. To address this challenge, we conducted a photograph-survey study of a greenway to examine the relationship between place preference, perceived health benefit, and environmental perception. Through a set of linear regression analysis, we found that: place preference is significantly and positively associated with six specific perceptions, including relaxation when walking alone, cheering of one’s mood, being away from daily life, traffic safety, recovery from stress, and mental fascination. Furthermore, we identified the important environmental perception elements that have significant positive or negative associations with each identified perception; these were carefully planned. This study is an initial effort to examine a critical urban land-use issue: appropriate planning of greenways in the city to promote public health and well-being. The research findings provide strong and clear guidance on planning strategies for urban greenways and shed light on future studies.

## 1. Introduction

Rational land use and sustainable development are the means to solve the problem of urbanization; the rationality of land use plays a restrictive role in urban planning. To solve the problem of land use, improving urban land-use efficiency and optimizing urban green-space planning are unified. It is necessary to conduct a comprehensive study of land use, due to the multiple causes of land-use change and the complexity of its problematic structure. The spread of COVID-19 made a large number of indoor public spaces no longer safe and healthy. Hence, the greenways in their living city became the main places for leisure and relaxation.

Improving land utilization efficiency can promote the sustainable development of cities. For urban residents, land utilization efficiency is affected by the degree of preference. Urban health include psycho–physical health and social health impacts were considered when assessing the impact on urban social status. The construction of urban greenways should focus on “green” and “health”. Information about the perceptions and attitudes of people, from landscape evaluation and preference studies, serves as an important scientific basis for the planning and management of an urban green space [1].

In the process of using green space, all activities will be linked to each other, thus forming a broader and richer range of activities overall. When a green space is preferred by people, it is used for a longer time; so, then it causes the activity to be a richer experience, and the land utilization efficiency is higher. In the background of urbanization, to solve the urban environmental problems from the perspective of urban green-space planning, we should improve the land utilization efficiency, and improve the degree of people’s place preference. 

### 1.1. Place Preference

We need to understand people’s place preference in relation to green space. Urban residents’ choice of green space is mainly based on subjective feelings, and the evaluation of the space is intuitive and perceptual; their brains and bodies respond positively to the landscapes that they prefer [2,3]. Studies have suggested that preferred landscapes are often restorative landscapes. Tree-lined streets and Savannah-like landscapes are highly preferred compared to other types of environments [4,5,6], and these landscapes also aid stress recovery [7,8,9] and provide restoration of one’s attention [10,11]. Through the spatial analysis of high-density urban greenways, this study looks for a way to optimize the spatial planning of urban greenways.

### 1.2. Perceived Health Benefits and Land Use

By creating subtle points, the green space can be extended to the greatest extent in terms of its use. Considering the increase in the existing urban population density and the abundance of indoor entertainment and leisure places, the utilization efficiency of greenways is an important indicator of their role. The utilization efficiency of greenways is related to the spatial planning, geographical location, and coverage area of the greenway [12,13], but it is also influenced by people’s subjective preference for it. This study explored the significant correlation between residents’ preferences and environmental perception factors with health recovery, aimed at improving urban land utilization efficiency and promoting residents’ health and urban health.

In China, the greenway typically adjoins a park or the urban outskirts close to a natural conservation area due to two major reasons. First and foremost, there have been very limited areas available for greenways with the urbanization in China. Second, the users of greenways prefer a more authentic greenway located in real nature. Quantifying and characterizing urban leisure space and leisure activity patterns reveal the spatial distribution of leisure resources and people’s behavior preferences. It can provide tailor-made guidance for reasonable urban resource allocation, space form design, and green sustainable development [14]. Because public urban green spaces have different characteristics and social uses within cities, it is furthermore important to assess people’s preferences about public urban green spaces. In fact, a better understanding of the preferences of a given city’s residents for their public green spaces may inform policy makers and city planners to effectively provide and manage urban green spaces to meet users’ needs [15]. The research on the degree of influence of urban parks on preferences will help the future construction of urban parks to better meet the needs of the public, enhance the park ecosystem services, and promote sustainable urban development [16].

### 1.3. Urban Greenways Construction

The ecology benefits of urban greenways have been the top concern for decades, which is often defined as a critical strategic ecological infrastructure [17]. In the rapid developing cities, such as those in China, greenway planning on a regional scale has largely neglected local citizens’, especially deprived citizens, basic needs for gaining health through the usage of nearby nature [18]. The greenway is also required to be safe and comfortable by maintaining users’ “social distance” [19].

Among the examination of the impacts of greenways at a human dimension, much attention has been given to physical activity, recreation [20], air pollution [21], etc. The mental health benefits of urban green spaces have gained more and more attention from scholars because of its rising importance in the field of public health, urban planning, and landscape architecture [22,23,24]. The mental health benefits from contact with nature mainly include mental fatigue recovery [8,25,26], mental stress reduction [7,27], increase in social cohesion [28], and promotion of mood [29,30]. However, scientific evidence directly found in the context of greenways in the highly dense urban areas is still sparse. In recent years, the functions of greenways in Chinese daily life are becoming more and more abundant. At the same time, there is a more complex planning trend in the construction of greenways [17]. The existing planning lacks consideration of users’ feelings. Some researchers suggest that more consideration should be given to path width, location, infrastructure, greenway pavements, and humanistic care in greenways’ planning [31]. Therefore, this study aims to explore the influencing factors of users’ mental health and greenways environment in Shenzhen, China; thus, discovering the fundamental factors to improve the utilization efficiency of urban green space.

### 1.4. Strategies for Improving Place Preference: Multilevel Mapping Model

Arguments are commonly made that sustainability challenges cannot be addressed effectively using conventional approaches to policy and planning. However, the existing preference promotion methods are imitation research based on the greenway characteristics of the ones with better indicators, or repetitive modeling and evaluation research. They have the disadvantages of poor universality and low solution efficiency after the location change, and have not formed a systematic optimization and specification process. The main reason is that they have not conducted fundamental traceability research from the formation mechanism of preference.

In order to realize the quantitative representation of people’s subjective feelings and ideas, and to characterize and calculate their relationship with the characteristics of the greenway environment, it is necessary to establish a mapping relationship model for numerical simulation. The purpose of establishing the framework of the interaction model is to carry out the numerical simulation of preference. The purpose of the numerical simulation is twofold. One is to predict the preference under different input conditions of environmental characteristics, so as to verify the advantages and disadvantages of the existing greenways. The other is to obtain the maximum preference, so as to realize the reverse design of greenway conditions through multiple groups of numerical simulations and comparisons, so as to obtain the optimal design parameters. A large quantity of sample data are obtained from field surveys to solve the established impact model framework and establish a greenways evaluation system with high realism based on the model. SPSS is the research tool and specific execution platform for model input, calculation, output, and analysis. However, there are many influencing factors on greenway preference and there is correlation and interaction between the factors. If the interaction relationship between the factors is ignored and only the cross-level direct transfer model between greenway environmental perception and place preference is established, the simulation deviation is large. Therefore, it is necessary to establish a multilevel mapping model. In this study, a multilevel mapping model of place preference, perceived health benefit, and environmental perception is established to improve the fitting accuracy of the actual complex impact relationship.

## 2. Methodology

This research built an evaluation system aiming at the greenway preference. The constructed system was to consider perceived health benefits and environmental perceptions as the two main categories of influencing factors. Hence, two major research questions were introduced. Question one: whether and to what extent people’s preference of greenway settings were associated with specific perceptions of the settings? Question two: Whether and to what extent those specific perceptions were associated with specific landscape space characteristics?

**Figure 1:**
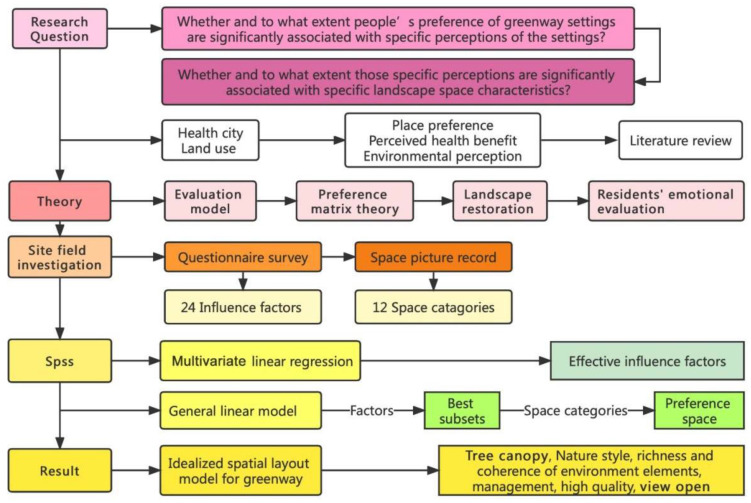
The research framework.

Due to the numerous influencing factors and their complex interactions, it was difficult to obtain the significance of these factors through repetitive experiments and analytical algorithms. Numerical simulation is a better method for establishing the greenways evaluation system. However, it is difficult to ensure the accuracy of the results by simply using the single-level mapping relationship as the basic framework of the evaluation system [32,33]. Therefore, a multilevel mapping model of perceived health benefits, environmental perceptions, and greenway preferences should be constructed, which can simulate the actual influencing mechanism with high accuracy [34]. The sampled and transformed quantitative data representing environmental perception factors and perceived health benefits were input and calculated in the greenways evaluation system. Then, the output results could be analyzed and used as a guide for input optimization. In this study, professional statistical analysis software and a proposed prediction algorithm were used to calculate and simulate a multilevel mapping relationship. By this means, the weight of each influencing factor was obtained through changing the initial settings of specific variables. The research framework is shown in Figure 1.

### 2.1. Research Design

Based on the needs of citizens, the frequency of use, space type, accessibility, attractiveness, restoration, recovery ability, vegetation coverage, biodiversity, and other aspects, this paper comprehensively discusses the spatial evaluation framework of urban greenways, and puts forward the index system of urban greenways spatial evaluation in combination with the theory of urban sustainable development.

In this study, we analyzed the correlation of 24 influence factors in the survey. A total of 60 photos were selected from 200 original photos for research using the Delphi method [35], and were used for correlation analysis. Finally, 24 questions and 60 photos were randomly combined into a questionnaire to collect the psychological and physical feedback of greenway users in Shenzhen. The collected questionnaire data were analyzed, and the analysis results provided optimization guidance for how current greenway planning affects the use preferences of greenways in Shenzhen.

**Figure 2:**
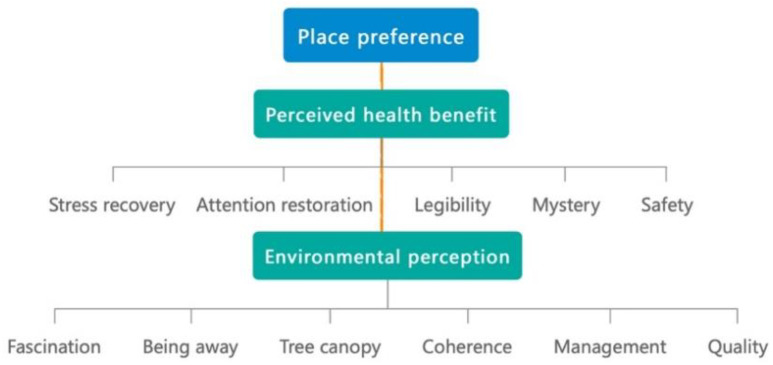
The classification of the influencing factors of greenway place preference.

In the online investigation, there were 35 questions in an online research questionnaire. A total of 24 questions dealt with the random mixing of greenway-optimization influencing factors. The 24 questions in each questionnaire were randomly combined with 60 photos. The 60 photos, and the other 11 questions dealt with the interviewers’ personal information and suggestions on the current situation of Shenzhen greenways. The answers to the questionnaires were divided into seven categories. The scale of level one to seven is used to represent the seven options, that include “extreme”, “moderate”, “slight”, “neutral”, “slight”, “moderate”, and “extreme” for each statement. The 1st to 3rd answers are negative options, and the 4th is an intermediate value option, and the others are positive options. The classification of the influencing factors of greenway place preference is shown in Figure 2.

### 2.2. Location of the Case Study

Shenzhen is one of the first cities equipped with urban greenways. Shenzhen is the most crowded city in China, and ranking fifth in the world’s highest density cities [36]. The exploitable land and living spaces in Shenzhen are becoming increasingly limited. Therefore greenways, as parts of the main green land, have high research value [37].

When choosing representative greenways land in Shenzhen, three factors were considered: population density; residential activity; and the surrounding environment [38]. The Qiaocheng East Road and Xiangmei Road were selected in this study. The Qiaocheng East Road, located in the Nanshan District, is on the first stage of the Shenzhen Greenway Development Project in 2009. The surrounding area of the Qiaocheng East Road has a high population density and a high frequency of residential activities. This road represents the history and development of Shenzhen’s greenways. Xiangmei Road is located in the center of the Futian District, with residential, commercial, and comprehensive land as its primary functions and consistently high activity. Qiaocheng East Road and Xiangmei Road are close to densely populated areas. The selections of the two roads are based on their heavy usage, long distance, and large radiation. The selected two main greenway planning guiding roles in optimizing the greenways in Shenzhen.

### 2.3. Photographs

In the field investigation stage of the study, a total of 6 h each day for 30 days, Monday through Friday, was spent photographing the subject area in clear, sunny conditions. A 130-degree lens was selected to take the Shenzhen greenway photographs, and the same aperture was maintained for consistency. At the process of taking the photographs, signposts, road signs, directional signs, people, bicycles, and passing animals were avoided whenever possible. The photographs were taken every 50–100 m passing through the greenway and keeping the greenway at the center of the photographs without any plants obscuring the view. In the process of taking photos, pedestrians, bicycles, and passing animals were avoided by blocking the surrounding physical environment as much as possible.

The impact of plants on land use is based on field measurements of the distance and direction between trees and residential buildings [39]. Based on three environmental elements (space scale, structure, and plant level), 60 greenway site photos were divided into 12 spatial types. The spatial scale was distinguished by front or back streets, landscape elements were distinguished by the different types of structures, and plant level was distinguished by tree, shrub, and grass levels. The Shenzhen greenway space category diagram is shown in Table 1.

### 2.4. Environmental Perceptions

Studying prior work by Blumentrath and Tveit, a landscape evaluation model was created, with influence factors including coherence, visibility, high quality and maintainability, naturalness, diversity, and accessibility [40]. Kaplan pointed out that there are two types of factors affecting place preferences, one is obvious and the other is inferred or predicted. Obvious factors include coherence and complexity, while inferred or predicted factors include readability and mystery [41]. Some studies have considered the likeability of urban landscapes as the basis for the optimization of urban image and emphasized that the internal significance of shaping urban image and imagination has a guiding impact on human behavior. Internal meaning affects their behavior, whether users go to a place, and how to go to a place [42,43]. Based on the analysis of the above scholars’ theoretical elements, this study selects the following elements as the perceived health-benefit factors and environmental perception factors in the high-density urban greenways spatial evaluation system: “coherence of environment elements” (CEE); “mood cheering” (MC); “prediction” (Pred); “mystery” (M); “fascination attraction” (FA); “richness of environmental elements” (REE); and “preference” (Pref).

The results of a landscape restoration research by Kaplan showed several key elements of restorative landscapes: distance; charm; continuity; and compatibility. The environment elements corresponding to these factors include environment brightness [44]; crown shape [45]; natural style; and environmental compatibility [46,47]. In addition, the tree distance, direction to building, climate region, leaf type, and percentage cover of buildings and trees on the plot can affect the carbon emissions of surrounding buildings, thus affecting the ecological effect of the land [48]. The well-connected and pleasant open spaces can bring relief to people living in high-rise buildings in congested neighborhoods beset by meager green spaces and stressful urban living [49]. These influencing factors include physical environment perception and personal perception. The influencing factors are divided according to the components of green environment space, space openness, space style, bearing capacity of diversity function, and the impact of space environment on perception. According to these elements, this study takes “view blocking general” (VBG), “view blocking with shrubs and grass” (VBSG), “brightness” (B), “tree canopy percent” (TCP), “compatibility for exercise” (CE), “away from urban environment” (AUE), “away from daily life” (ADL), and “naturalness” (N) as the environmental perception elements in the urban greenway spatial evaluation system.

In the case study of established suburban communities, Lin proposed residents’ emotional evaluation and neighborhood visual elements; the significant impacts on the emotional evaluation elements are pavement shape, street facilities, and pavement texture [50]. Based on these research elements, this paper selects four environmental perception elements: “pavement quality” (PQ); “complexity of paving pattern” (CPP); “overall quality” (OQ); and “management” (Mana). Previous research on the impact of landscape variables on pedestrian safety has pointed out that overall safety and traffic safety are very important for city space planning [51,52]. In this study, “general safety” (GS) and “traffic safety” (TS) were selected in the spatial optimization evaluation system of urban greenway.

An observational study was conducted on students who felt slight pressures after a final exam. The research results showed that the group watching daily natural color slides had a better recovery effect than the group watching urban scenery without plants [23,53]. In the late 1990s, another scholar repeated the study and found that the group watching urban scenes without vegetation experienced an increase in pressure and a less positive impact. Directing people’s attention can train their thinking, set goals for rapid problem solving, initiate and execute tasks, self-monitoring, and regulation [53,54,55]. According to the research, this study considers three factors as the spatial optimization evaluation system for high-density urban greenways. They are “stress recovery” (SR), “attention restoration” (AR), and “relaxed or anxious if alone” (RAA). All of the impact factors are listed in Table 2.

### 2.5. Procedure

To identify the influencing factors, that is, the prominence of the components of greenway space, this study established two automatic linear models: one dealt with the relationship between place preference and perceived health benefits, and the other dealt with the relationship between perceived health benefits and environmental perception.

The main problem we faced was extracting the desired correlations between the indicators. In order to minimize possible attenuation and fatigue due to prolonged exposure to a single picture, the participants were asked to answer the 24 questions using a likability scale rating based on different pictures. Therefore, for each participant, his or her rating to each indicator was also influenced by the picture he/she was exposed to. To solve this problem, we collapsed all ratings by averaging the scores to a matrix of 24 indicators over 60 picture samples. Therefore, we were able to separate the influence from the pictures and we analyzed the interactions between picture stimuli and indicators when necessary.

**Figure 3:**
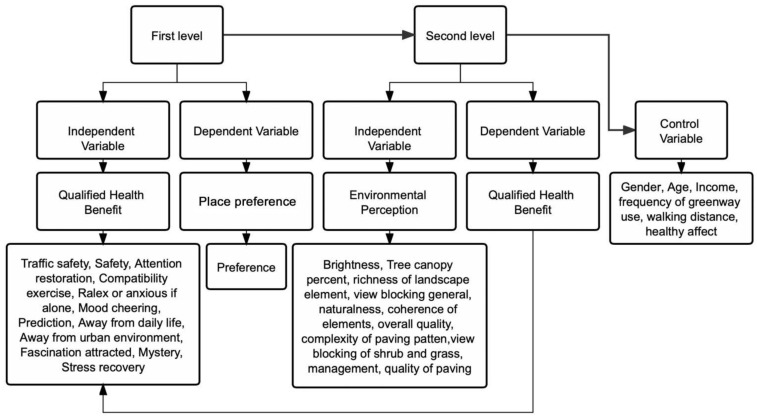
The multilevel mapping model.

After individual regression analysis for each group, the coefficient of the first layer was explained by high-level variables, and the regression coefficient of each group of the first layer was analyzed as the result variable of the second layer. In the first layer of the multilevel mapping model, qualified health benefit is the independent variable and place preference is the dependent variable. In the second layer, environmental perception is the independent variable and perceived health benefit is the dependent variable. The respondents’ gender, age, income, frequency of greenway use, walking distance to greenway, and healthy effects were taken as control variables. The multilevel mapping model is shown in Figure 3.

### 2.6. Statistical Analysis

After correlation analysis and multivariate linear regression analysis, the rationality of the theory and questionnaire factors in this study were verified. Using a general linear model analysis, this study determined the evaluation system based on 24 factors. The relationship among all of the factors was also clarified. This study used an automatic linear model analysis to establish a new likability result model, and combined it with the best subset from automatic linear model analysis to construct a better high-density urban greenway planning principle.

## 3. Results

According to the detailed analysis of the results, greenways’ quality management has a strong impact on respondents’ psychology. The quality and management degree of the greenways’ paving pattern also affects the guarantee of the greenways’ fitness function. The sight line and plants have a greater impact on the respondents’ sense of distance. Sight lines, plants, and quality control have a significant impact on relieving users’ pressure and restoring users’ attention and emotion.

A total of 1212 interviewees completed this research questionnaire survey, of which 87% contributed valid data. Most of the interviewees were young people, with the majority aged between 18 and 40, that can best represent most of the Shenzhen citizens. More than half of the interviewees have lived in Shenzhen for less than one year, and the vast majority were population that originated from outside Shenzhen, who included businessmen, student teachers, company staff, labor, retirees, the unemployed, civil servants, service providers, general service providers, and other professionals. Regarding monthly income, as there were many students among the interviewers, many of the interviewees earned less than CNY4000, and more than half of them earned less than CNY8000. Most of the interviewees lived in communities not far from the city greenways; only a small number of interviewees were living in communities over 1500 m to the nearest greenway. Most people used the greenway, although not very often. According to basic data analyses, the interviewees’ main reasons for using the greenway were walking, passing through, resting, and running, and the main function of riding along the greenway was not very prominent. Most of the interviewees have no relevant professional background, and it implies that this research questionnaire survey was not directed towards any interviewee with a particular professional background, but rather all citizens. Only a small number of interviewees agreed that the Shenzhen city greenways have a negative influence on their health, which implies that the Shenzhen city greenways have an overall positive influence on public health.

### 3.1. Questionnaire

After sorting out all of the respondents’ suggestions, it can be seen that some respondents thought that some of the greenway space were too enclosed, while others thought that some greenway spaces were too open. Some respondents believed that allowing cyclists and pedestrians to use the same greenway increased the risk of accidents, so they suggested that safer designs should be implemented, such as widening the greenway. As pointed out in the “European Greenway Association” [56], the international scale of bicycle lanes ranged from 1 to 1.5 m. However, the widest bicycle-lane span of the Shenzhen greenways is 0.6 to 0.9 m, and only a few parts reach 0.9 to 1.1 m. In other words, it is very important to widen the Shenzhen greenways.

**Figure 4:**
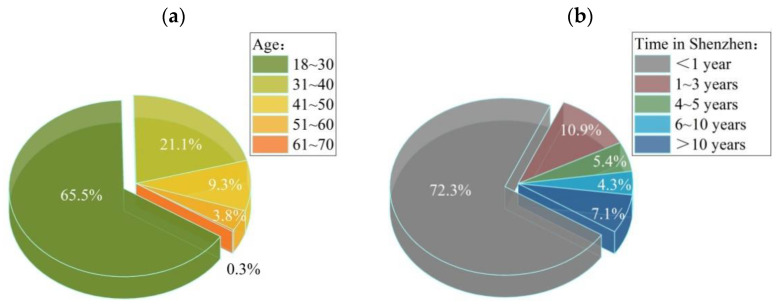
Composition of the interviewees: (**a**) Interviewee age composition; (**b**) Living time of the interviewees in Shenzhen.

**Figure 5:**
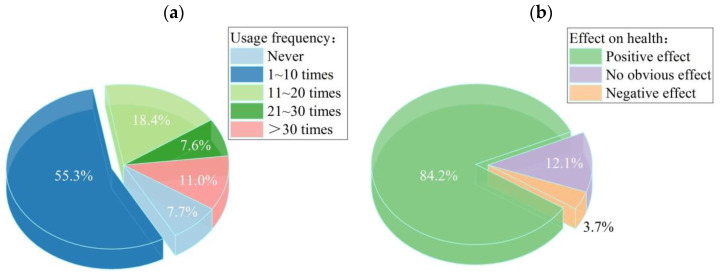
Background results: (**a**) Interviewee usage frequency; (**b**) Greenway effects on health.

By analyzing the questionnaire data, the following results were obtained: the main function of the respondents using the greenway was walking and passing; the secondary function was resting and running; and the lack of riding on the greenway was not significant. Generally speaking, the function of people using the greenway has changed from riding and walking, to leisure, fitness, and rest. With regard to the impact of greenways on residents’ health, 84.2% of the respondents believed that Shenzhen greenways could alleviate life pressures and have positive impacts on their physical and mental health, against 12.1% who were neutral about it, and 3.7% who opposed it. In general, Shenzhen greenways have a positive impact on urban health. The composition of the interviewees is shown in Figure 4. Greenway usage frequency and effects on health degree are shown in Figure 5a,b for details, respectively.

The respondents’ gender, age, monthly income, walking distance from residential area to nearest greenway, the frequency of using the greenway every month, and the impact on health were taken as the control variables for the correlation of influencing factors. The multivariate linear regression analysis report is shown in Appendix A.

### 3.2. Main Finding

The main findings of this study are as follows:

1. There are six most significant influencing factors (best subset) of “perceived health benefits” on “place preference”. They are “relaxed or anxious if alone”, “mood cheering”, “away from daily life”, “traffic safety”, “stress recovery”, and “fascination attracted”, ranked from the most significant to less significant;
2. There are nine environmental factors that affect the above six perceived health benefits. According to correlation arranged as follows, “overall quality”, “richness of environmental elements”, “view blocking general”, “quality of paving”, “management”, “coherence of environment element”, “naturalness”, “tree canopy percent”, and “view blocking with shrubs and grass”.

A summary of the positive or negative correlations between each impact factor is presented in Table 3. The sorted results are listed on the right side of the table.

#### 3.2.1. Best Significant Influence Subset

There are six most significant influencing factors of “perceived health benefits” on “place preference”. It can be seen that “relaxed or anxious if alone” has the most significant effect on “place preference”, that is, people in the greenway are most concerned about their feeling of relaxation on the greenway. Meanwhile, “mood cheering”, “away from daily life”, “traffic safety”, and “stress recovery” affect “place preference” to a certain degree for sig.-values varying from 0.001 to 0.009. The diagnostic results are shown in Table 4.

**Figure 6:**
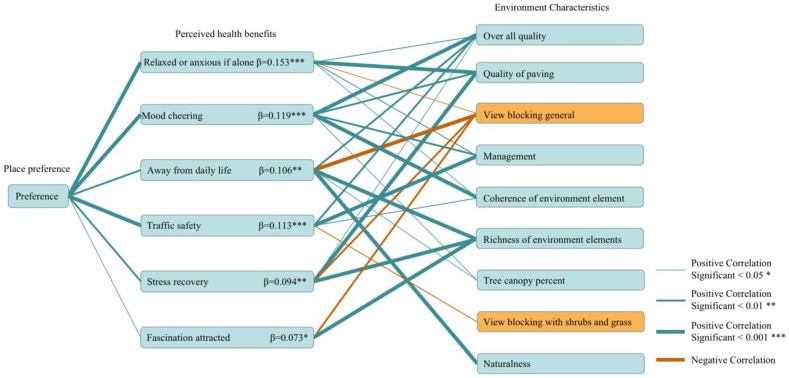
Direct correlation of place preference, perceived health benefits, and environment characteristic.

Subsequently, the relationships between the above six perceived health benefits and the corresponding environmental perceptions were also analyzed. First, with respect to “relaxed or anxious if alone”, the statistical results of the significance test on each factor are quality of paving, overall quality, view blocking general, management, and coherence of environmental element. “Quality of paving” make people feel more relaxed in the greenway. Second, with respect to “mood cheering”, the statistical results of the significance test on each factor are overall quality, coherence of environment element, management, quality of paving, and tree canopy percent. “Overall quality” and “coherence of environment element” could greatly cheer people’s mood in relation to the greenway. Third, with respect to “away from daily life”, the statistical results of the significance test on each factor are richness of environmental elements, view blocking general, naturalness, overall quality, and tree canopy percent. Fourth, with respect to “traffic safety”, the statistical results of the significance test on each factor are management, overall quality, view blocking of shrubs and grass, overall quality. Good greenway management could make people feel low risk of the greenway traffic safety to the greatest extent. “overall quality” have the same effect, but to a lesser extent. Fifth, with respect to “stress recovery”, the statistical results of the significance test on each factor are quality of paving, richness of environmental elements, view blocking general, and overall quality. Sixth, with respect to “fascination attracted”, the statistical results of the significance test on each factor are richness of environment elements and view blocking general. The richness of environmental elements in relation to the greenway could attract people’s fascination. View open has the same effect, but to a lesser extent. The statistical results of the significance for each factor are shown in Figure 6.

#### 3.2.2. Significant Spatial Category

Combined with the above six perceived health benefit factors and measures, the influencing factors of different types of greenway spaces and rating systems are regressed to find the optimal space category of high-density urban greenway spaces. The results of this spatial category can be used as the optimization principle for high-density urban greenways spatial planning. The analysis process was divided into two steps.

The first step was to determine the most prominent spatial category corresponding to the six best subsets. Greenway spaces of a spatial category 6 and 8 have a positive impact on people’s perceived health benefits, while spatial category 7 and 12 have a negative impact on people. The results show that people do not like structures around the greenways, especially in the frontage street, such as railings, stairs, overpasses, and viaducts. The second step was to find the controversial space category. The results show that spatial category 3, 10, and 11 were controversial positive spaces. The greenway space with planted trees, shrubs, short grass in frontage streets and built structures in back streets made people feel nervous and unhappy. When people walk alone in spatial category 1 and 2, they feel relaxed, mood cheering, and relieve some of their anxiety. The space category significance and main findings are listed in Table 5.

**Figure 7:**
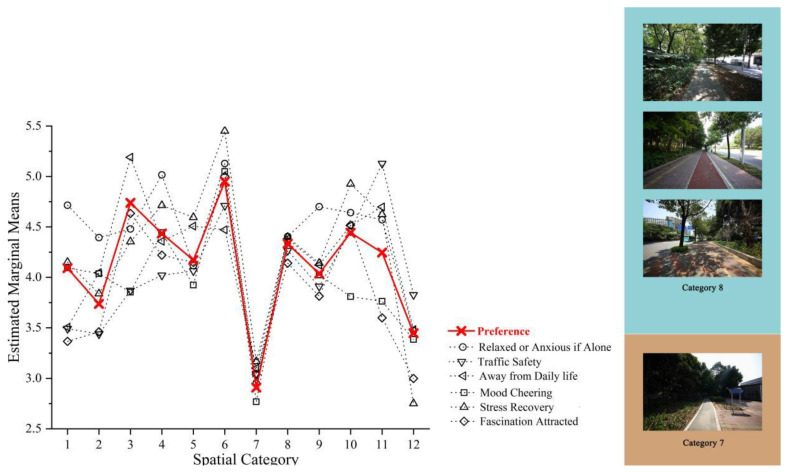
The data offset rate of estimated marginal means of spatial category.

The comparison between the best subset result and the place preference result shows that the data offset rate of spatial category 7 and 8 is between 0.6–8.9%, which is significantly smaller than that of other elements by 0.9–24.7%. Hence, the evaluation of the positive and negative effects of spatial category 8 and 7 is the least controversial. It also proves that people prefer the space with trees in the frontage street and planted trees, shrubs and short grass in the back street, while they do not like trees and structures mixed in the frontage and back streets of the greenways. The correlation values between the best subset and place preference are shown in Figure 7.

## 4. Discussion

One of the main findings is that people prefer the greenway space with a high proportion of tree canopy coverage and they also prefer rich plant elements of trees, shrubs, and short grass on the back street. Meanwhile, people doesn’t like railings, overpasses, walls, and structures in their view. For Shenzhen, with a subtropical climate, the Shenzhen greenways’ design needed increased shelter space. It also required a design of the trees to create a “half private-half public” space environment, but to keep the users’ views open, and can view through the lower edge of tree canopy. It also required a minimal use of shrubs, or the use of shorter shrubs, increased use of mixed ground-cover plants and short grass to create a rich plant wilderness hillside in the high density city center area. This wilderness hillside looks like a wilderness, but each plant, road, and facility have all been designed and well managed.

Based on different categories of user, the physical characteristics of different users are different, so each appropriate distance landscape point should be set up to provide some rest areas, pavilions, and rain shelters which have the function of blocking rain. This means that the user can rest more comfortably and spend the waiting time. On analysis of the interviewers’ Shenzhen greenway advice, the results show that management is very important, because road sign deletion, inadequate emergency services, severe damage to the paving pattern will greatly increase the greenway safety risk. One the one hand, the greenways need a complete greenway identification system; on the other hand, there is also a need to standardize and correct the greenway expression identification system, to guarantee that the information provided on the greenway is adequate and accurate, in order to avoid the user encountering unknown safety risks when walking the wrong way.

### 4.1. Contributions and Implications

Under the influence of COVID-19, the role of ground urban greenway has become increasingly prominent. Therefore, to increase people’s use of urban greenways, which depends on people’s preference for greenways, it is necessary to explore the perspective of the most fundamental influence mechanism to find out the influencing factors and interaction relationships of people’s liking for greenways, thus, the reverse design of the greenway is carried out to make the greenways more popular under the condition that the effect of the greenways on people’s health is not reduced. After analysis, the greenway impact factors are not simple direct mapping relationships, but have multilevel progressive impact relationships. Therefore, it is necessary to simulate this relationship by establishing a multi-subset mapping model, and implement the algorithm flow through professional analysis software. In this paper, the independent variable set described in this paper includes the persistent health benefit of the greenways, and finally achieves the goal of trace-ability reverse design to increase the popularity of the greenways by optimizing the environmental characteristics of the greenways.

**Figure 8:**
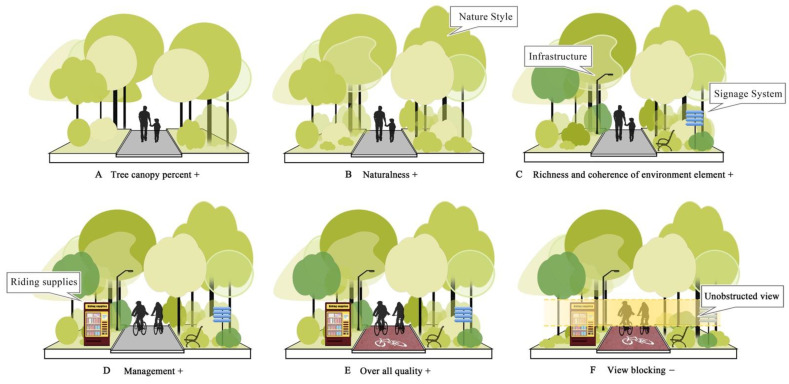
Idealized spatial layout model for greenway. (**A**) Add tree canopy; (**B**) Nature style; (**C**) Richness of plants and increase infrastructure; (**D**) Well managed; (**E**) High quality space; and (**F**) View open.

The huge overpass at the intersection of urban trunk roads makes people feel extremely unfavorable to the passage of ground greenway. The transition between the greenway and the surrounding environment is important. That is consistent with the research results; the greenway space with open lines of sight is more popular. According to the survey, it was found that the structures do not appear in the users’ favorite spaces, which is consistent with the feedback of the questionnaire. Idealized spatial layout models for ground greenways are shown in Figure 8.

This research studied the factors that influence urban greenway environments; these factors influence the users’ personal safety, mental health, and physical health in the urban area. The study also found the greenway design method promoted users’ personal safety, mental health, and physical health in urban areas. The types of greenways with overall good quality, especially the quality of greenway paving, including the management and maintenance of the greenways, these influencing factors are the great discoveries of urban planning. The people’s preference for dense tree canopy cover, but also an open view, in particular, no view blockage caused by shrubs and short grass are the important findings of this research.

Regarding the analysis of the space category, trees, shrubs, and grasses at the frontage street, with trees at the back street (category 6); trees at the frontage street and trees, shrubs, and grasses at the back street (category 8), are categories more suitable for urban greenway space planning. Prediction and ability to view open space without structures were most valued by users. As research, the best high-density city greenway space is one with a more open space, with the user’s view more open, and with the most tree canopy coverage, so that the whole landscape environment is more natural and is well managed with a good paving pattern. Open space could let the user feel safe; increasing the tree canopy could let the user have a cooler environment in summer; reducing the shrub coverage could let the user have an open view and also feel safe. Designing a more natural greenway environment could let the user feel that they are away, and both being well managed and a simple paving pattern could reduce the risks of greenway traffic accidents.

### 4.2. Limitations and Opportunities for Future Research

This study constructs a new effectiveness framework for future related research. These findings are very important and could potentially fill the landscape research gap. This study proposed a greenway space optimization method. The new method is not only applicable to the optimization of the greenway, greenway optimization is just the particular focus of this paper. The proposed method with high universality is also applicable to the traceability reverse optimization design of parks, public fitness places, and children’s playgrounds. It fills a part of the gap in greenway space design and research, and improves the space evaluation system.

The study focuses on the analysis of city spaces, plants, and people’s mental health. Traffic safety, such as the contradictions between the traffic and pedestrians; traffic conflicts between motor vehicles and bicycles; conflicts between pedestrians and motor vehicles, all need to be discussed further. The impact of the environmental voice, like the motor vehicle voice, on pedestrian health also needs to be discussed further. The optimization of urban spatial planning has a positive impact on the healthy development of residents, and accelerates the ecological construction of cities.

## 5. Conclusions

Urban greenways bring considerable benefits to urban land, but are they a preferred place to relieve users’ psychological pressure? This research is a new discovery in urban greenways, and filled a research gap in urban planning and research. This research conclusion can positively affect the land use efficiency, reduce the pressure on urban land development, and enable the city to enter a stable period of sustainable development in the future.

We used multilevel mapping research method to complete a basic traceability research from the formation mechanism of preference. This study established a multilevel mapping model of place preference, perceived health benefits, and environmental perception. We found that there are many factors affecting greenway preference, and there were correlations and interactions among the three levels of factors. This model improved the fitting accuracy of the actual complex impact relationship and it was effective.

Further, the greenway’s effects need richness of plants and harmonious environmental elements with natural style. Researchers should further explore the relationship among place preference, environment elements, and human mental health. More open views, more open paving patterns, and increased simplicity overall also contributed to a better greenway. This conclusion could resolve problems that arise in the midst of high-density residential areas and limited living land.

This paper brought a new research perspective and provides a new urban planning optimization strategy for improving urban land utilization efficiency. The research results were in line with the research theme, that was, to promote residents’ preferred place, and to coordinate with the healthy development of the city at the same time. This paper studied the spatial elements of urban green space, solved the contradiction of land development and utilization with “less land and more people”, and fully and reasonably optimized all of the land resources that can be developed and utilized, so as to improve the land utilization efficiency of urban green space.

## Figures and Tables

**Table 1:** SZ greenway space categories.

Greenway Space Category			
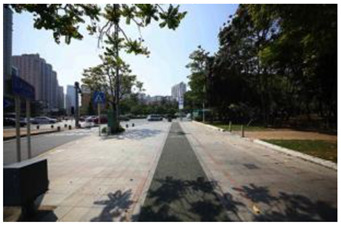	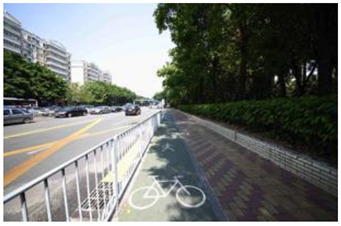	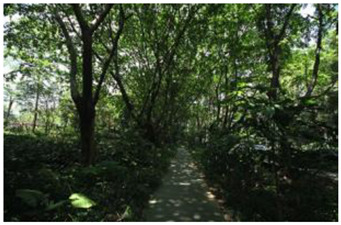	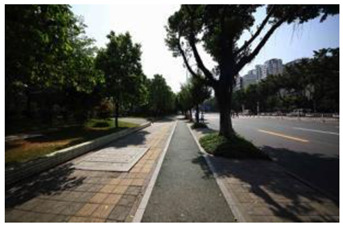
1 BS: tree	2 BS: shrub/grass	3 FS: tree/shrub/grass BS: tree/shrub/grass	4 FS: tree BS: tree
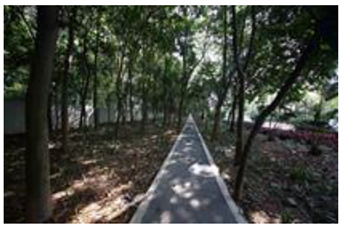	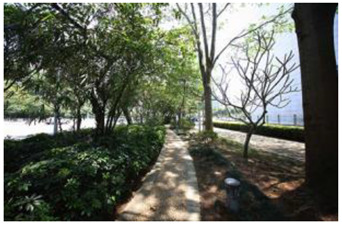	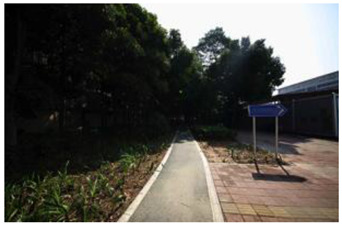	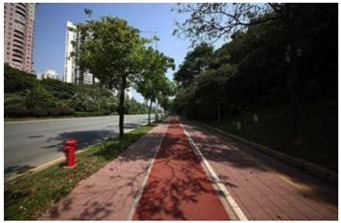
5 FS: tree BS: tree/built structure	6 FS: tree/shrub/grass BS: tree	7 FS: tree/built structure BS: tree/built structure	8 FS: tree BS: tree/shrub/grass
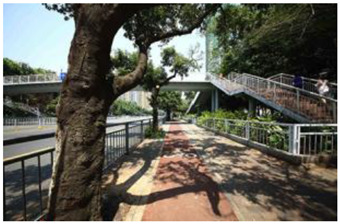	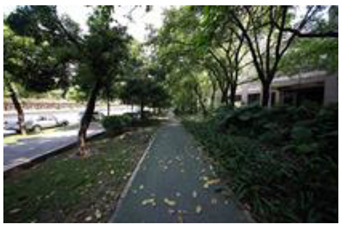	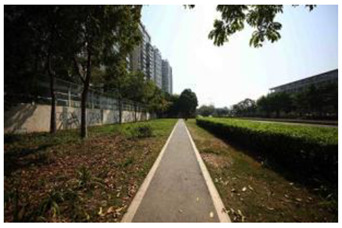	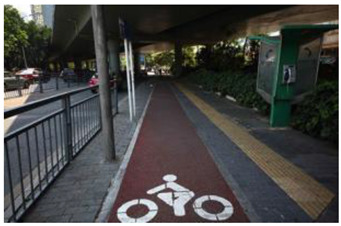
9 FS: tree BS: built structure	10 FS: tree/shrub/grass BS: built structure	11 FS: shrub/grass BS: built structure	12 FS: built structure BS: shrub/grass

FS represents frontage street; BS represents back street. The 12 categories: back street with trees (Category 1); back street with shrubs and grasses (Category 2); frontage and back street with trees, shrubs, and grasses (Category 3); frontage and back street with trees (Category 4); frontage and back street with trees, and back street with built structure (Category 5); frontage street with trees, shrubs, and grasses, and back street with trees (Category 6); frontage and back street with trees and built structure (Category 7); frontage street with trees, shrubs, and grasses at the back street (Category 8); frontage street with trees, and built structure at the back street (Category 9); frontage street with trees, shrubs, and grasses, and built structure at the back street (Category 10); frontage street with shrubs and grasses, and built structure at the back street (Category 11); frontage street built structure, and back street with shrubs and grasses (Category 12). Each category is represented in the following photographs.

**Table 2:** Influencing Factors.

Researcher	Theory	Attribute	Influence Factor
C. Blumentrath and M.S.Tveit Stephen Kaplan	A model of landscape elements Preference matrix theory	Coherence Legibility Mystery	Coherence of environment element (CEE) Mood cheering (MC) Prediction (Pred) Mystery (M) Fascination attracted (FA) Richness of environment elements (REE) Preference (Pref)
Stephen Kaplan	Restoration of landscape	Fascination Extent (Coherence) Compatibility Being away	View blocking general (VBG) View blocking of shrubs and grass (VBSG) Brightness (B) Tree canopy percent (TCP) Compatibility for exercise (CE) Away from urban environment (AUE) Away from daily life (ADL) Naturalness (N)
Shih-Hsien Lin	Affective appraisal of residents and visual elements in the neighborhood	The pavement pattern The street furniture The pavement texture	Quality of paving (QP) Complexity of paving pattern (CPP) Overall quality (OQ) Management (Mana)
Rosenblatt Naderi	The effect of landscape variables on pedestrian health and safety	Landscape general safety	General safety (GS) Traffic safety (TS)
Ulrich and Parsons	Natural environment is the key component to human response	Stress reduction	Stress recovery (SR) Attention restoration (AR) Relaxed or anxious if alone (RAA)

**Table 3:** Automatic linear models result (*, **, *** represent significance with 0.05, 0.01, and 0.001, respectively; +, − represent positive and negative correlation, respectively).

Likability	Perceived Health Benefits	Environmental Perception	Result
	Significant Best Subset Factors	Significant Best Subset Factors	
Pref	RAA (+) ***	QP (+) *** OQ (+) * VBG (−) * Mana (+) * CEE (+) *	Overall quality + Richness of environment elements + View blocking general − Quality of paving + Management + Coherence of environment element + Naturalness + Tree canopy percent + View blocking with shrubs and grass −
	MC (+) **	OQ (+) *** CEE (+) *** Mana(+) ** QP (+) ** TCP (+) *	
	ADL (+) **	REE (+) *** VBG (−) *** N (+) *** OQ (+) ** TCP (+) *	
	TS (+) **	Mana (+) *** OQ (+) ** VBSG (−) * CEE (+) *	
	SR (+) **	QP(+) *** REE (+) *** VBG (−) ** CEE (+) *	
	FA (+) *	REE(+) *** VBG (−) **	

**Table 4:** The multilevel mapping model results (+, − represent positive and negative correlation, respectively).

Effect on Place Preference	Perceived Health Benefits	RAA			MC			ADL			TS			SR			FA		
		β		sig	β		sig	β		sig	β		sig	β		sig	β		sig
		0.153		0.000	0.119		0.001	0.106		0.003	0.113		0.001	0.094		0.009	0.073		0.039
**Corresponding** **Environmental** **Perceptions**			**β**	**sig**		**β**	**sig**		**β**	**sig**		**β**	**sig**		**β**	**sig**		**β**	**sig**
		**QP**	0.138	0.000	**OQ**	0.171	0.000	**REE**	0.146	0.000	**Mana**	0.148	0.000	**QP**	0.145	0.000	**REE**	0.194	0.000
		**OQ**	0.095	0.013	**CEE**	0.148	0.000	**VBG**	−0.144	0.000	**OQ**	0.124	0.001	**REE**	0.134	0.000			
		**VBG**	−0.089	0.012	**Mana**	0.107	0.003	**N**	0.125	0.000	**VBSG**	−0.086	0.014	**VBG**	−0.120	0.001	**VBG**	−0.115	0.001
		**Mana**	0.080	0.032	**QP**	0.106	0.003	**OQ**	0.115	0.001	**OQ**	0.089	0.015	**OQ**	0.085	0.019			
		**CEE**	0.075	0.041	**TCP**	0.078	0.021	**TCP**	0.046	0.021									

**Table 5:** Space category signification and main finding.

Relationship	Factor	Category			Most Prominent Category	Controversial Category
Preferred category	Relax or anxious if alone	4	6	8	6, 8	3, 10
	Mood cheering	4	6	8		
	Away from daily life	3	10	11		
	Traffic safety	6	11			
	Stress recovery	6	8	10		
	Fascination attracted	3	6			
Dislike category	Relax or anxious if alone	12	7		7, 12	1
	Mood cheering	7	12			
	Away from daily life	1	12	7		
	Traffic safety	7	2	1		
	Stress recovery	12	7			
	Fascination attracted	12	7			

## Appendix A

**Table A1:** Multivariate linear regression analysis report (* and ** represent the correlation which are significant at the 0.05 level and 0.01 level (2-tailed), respectively).

	**RAA**	**Pred**	**M**	**FA**	**PQ**	**VBG**	**N**	**ADL**	**REE**	**TS**	**Mana**	**CEE**	**AUE**	**OQ**	**MC**	**Pref**	**CPP**	**CE**	**AR**	**GS**	**B**	**SR**	**TCP**	**VBSG**
**RAA**	1																							
**Pred**	0.396 **	1																						
**M**	−0.303 *	−0.649 **	1																					
**FA**	0.233	−0.130	0.261 *	1																				
**PQ**	0.334 **	0.464 **	−0.320 *	0.187	1																			
**VBG**	0.417 **	0.679 **	−0.687 **	−0.042	0.507 **	1																		
**N**	−0.143	−0.485 **	0.653 **	0.368 **	−0.230	−0.502 **	1																	
**ADL**	0.118	−0.321 *	0.422 **	0.376 **	−0.159	−0.225	0.436 **	1																
**REE**	0.151	0.057	0.118	0.451 **	0.050	−0.127	0.236	0.256 *	1															
**TS**	0.193	0.264 *	−0.109	0.161	0.108	0.231	0.064	0.203	−0.126	1														
**Mana**	0.375 **	0.452 **	−0.401 **	0.111	0.612 **	0.409 **	−0.367 **	−0.118	0.013	0.094	1													
**CEE**	0.494 **	0.445 **	−0.344 **	0.322 *	0.430 **	0.426 **	−0.083	0.104	0.250	0.327 *	0.475 **	1												
**AUE**	0.030	−0.335 **	0.532 **	0.569 **	−0.044	−0.235	0.510 **	0.553 **	0.183	0.307 *	0.014	0.207	1											
**OQ**	0.394 **	0.443 **	−0.234	0.393 *	0.552 **	0.424 **	0.064	0.187	0.302 *	0.231	0.456 **	0.544 **	0.179	1										
**MC**	0.536 **	0.474 **	−0.412 **	0.191	0.433 **	0.483 **	−0.176	0.054	0.165	0.250	0.406 *	0.515 **	0.144	0.485 **	1									
**Pref**	0.355 **	0.107	0.069	0.363 **	0.241	0.051	0.209	0.417 **	0.372 **	0.329 *	0.265 *	0.392 **	0.508 **	0.332 **	0.363 **	1								
**CPP**	0.289 *	0.257 *	−0.331 **	0.081	0.304 *	0.299 *	−0.185	−0.243	−0.121	0.071	0.397 **	0.182	−0.080	0.221	0.219	0.114	1							
**CE**	0.368 **	0.082	−0.006	−0.011	0.355 **	0.173	0.016	0.305 *	−0.113	0.191	0.275 *	0.255 *	0.167	0.235	0.286 *	0.321 *	−0.106	1						
**AR**	0.375 **	−0.147	0.197	0.536 **	0.296 *	−0.030	0.402 **	0.501 **	0.328 *	0.169	0.171	0.332 *	0.527 **	0.487 **	0.350 **	0.487 **	0.000	0.335 **	1					
**GS**	0.461 **	0.614 **	−0.707 **	−0.124	0.390 **	0.705 **	−0.562 **	−0.237	−0.119	0.241	0.506 **	0.479 **	−0.316 *	0.471 **	0.515 **	0.048	0.413 **	0.258 *	−0.028	1				
**B**	0.252	0.533 **	−0.542 **	−0.060	0.324 *	0.640 **	−0.417 **	−0.239	0.065	0.105	0.338 **	0.339 **	−0.236	0.330 **	0.389 **	−0.082	0.239	0.223	−0.028	0.616 **	1			
**SR**	0.368 **	0.087	0.068	0.372 **	0.382 **	0.139	0.330 *	0.331 **	0.201	0.366 **	0.209	0.366 **	0.369 **	0.441 **	0.392 **	0.462 **	−0.038	0.393 **	0.601 **	0.123	0.123	1		
**TCP**	0.032	−0.450 **	0.583 **	0.324 *	−0.185	−0.514 **	0.708 **	0.355 **	0.116	−0.022	−0.274 *	−0.155	0.489 **	−0.054	−0.103	0.373 **	−0.091	0.046	0.353 **	−0.492 **	−0.592 **	0.299 *	1	
**VBSG**	−0.220	−0.564 **	0.698 **	0.229	−0.418 **	−0.720 **	0.697 **	0.341 **	0.232	−0.135	−0.250	−0.238	0.405 **	−0.166	−0.245	0.107	−0.227	−0.170	0.184	−0.641 **	−0.550 **	0.131	0.671 **	1

* Correlation is significant at the 0.05. ** Correlation is significant at the 0.01.
